# Evaluation of Factors which Influence Mortality in Gram-positive Bacteremia in Hemodialysis Patients

**DOI:** 10.7759/cureus.2917

**Published:** 2018-07-03

**Authors:** Kerry Anne Rambaran, Saeed K Alzghari, Charles F Seifert

**Affiliations:** 1 Clinical Sciences, Keck Graduate Institute, Claremont, USA; 2 Genomics, Gulfstream Diagnostics, Dallas, USA; 3 Pharmacy Practice, Texas Tech University Health Sciences Center School of Pharmacy, Lubbock, USA

**Keywords:** hemodialysis, vancomycin, vascular access, pre-dialysis

## Abstract

Vascular access infection is one of the major contributors to hemodialysis (HD) patient morbidity and mortality. There is a paucity of consensus guidelines on vancomycin use in the HD population. The primary objective of this study was to determine if vancomycin serum concentrations were associated with positive outcomes in HD patients with Gram-positive bacteremia. A retrospective cohort study conducted at a 443-bed tertiary teaching county hospital from January 1, 2010 to January 1, 2016 was performed. Patients aged 18-89, with chronic renal failure on hemodialysis who presented with positive blood cultures with Gram-positive bacteria and received intravenous vancomycin for at least 24 hours were evaluated. A multivariate analysis was utilized comparing factors related to outcomes including Simplified Acute Physiology Score II (SAPS II), loading dose, 30-day mortality and vancomycin serum concentrations. A total of 139 patients were obtained, 90 of whom had documented pre-dialysis serum vancomycin concentrations. A multivariate analysis showed that a lower SAPS II score [OR 1.220 (95% CI: 1.086-1.370, p < 0.0001)], a higher loading dose/kg [OR 0.7911 (0.6302-0.9929, p = 0.0239)], and pre-dialysis concentrations between 15 and 20 mcg/mL [0.05437 (95% CI: 0.0033-0.8891, p = 0.0099)] were associated with decreased mortality (overall multivariate model, p < 0.0001). When patient acuity and loading dosing are taken into account, pre-dialysis vancomycin serum concentrations between 15 and 20 mcg/mL were associated with decreased mortality in Gram-positive bacteremic intermittent HD patients. Further prospective studies are needed to assess whether targeting a pre-dialysis serum vancomycin concentration of 15-20 mcg/mL can improve mortality.

## Introduction

According to the United States Renal Data System (USRDS) 2016 annual report, 87.9% of incident end-stage renal disease (ESRD) patients initiated renal replacement therapy with hemodialysis (HD). While the rate of hospitalization in HD patients due to infection has decreased to 10.6% over the years, it remains a significant issue in this patient [1]. Infection, often related to vascular access, is one of the major contributors to HD patient morbidity and mortality, accounting for 9.5 to 36% of deaths [2-3]. Vascular access infections (most commonly found in patients utilizing dialysis catheters) are reported to be the source in up to 48 to 73% of all bacteremias in HD patients [4]. As such, antibiotic use in HD patients is not uncommon, particularly vancomycin, as it provides coverage against Gram-positive organisms inclusive of *Staphylococcus *and *Streptococcus *[5-6]; *Staphylococcus aureus* is reported as one of the leading causes of bacteremia, with a 1-year all-cause mortality of 62% and 5-year mortality of 72% [7].

Vancomycin is a glycopeptide antibiotic which has an elimination half-life of 5–11 hours in adults with normal renal function and 200–250 hours in patients with ESRD [8-10]. In non-ESRD patients, typical doses are calculated based on 15 mg/kg every 8 to 12 hours with a treatment target trough serum concentration of at least 10 mcg/mL or 15 to 20 mcg/mL in severe infections [11]. It is suggested that in order for HD patients to obtain a ‘trough’ serum concentration between 15 and 20 mcg/mL, a loading dose of approximately 20 mg/kg dosed may be required; however, there is no data to confirm these levels are substitute markers of the desired area under the curve (AUC) [12]. It should be noted that vancomycin is partially removed (approximately 30%–50%) by most of the currently available high flux HD filters [13-16].

Of note, Vandecasteele et al. reported an established linear relationship between AUC/MIC (minimum inhibitory concentration) and pre-hemodialysis vancomycin serum concentrations. They purported that for bacteria with an MIC of 1 mcg/L, pre-hemodialysis vancomycin serum concentrations of 10, 15 and 20 mcg/mL corresponded to the calculated 24 hour AUC/MIC of 269, 404 and 538 respectively in patients being dialyzed with high flux dialyzers. Thus leading to the conclusion that a pre-dialysis serum concentration of 15–20 mcg/mL or greater may be adequate to treat oxacillin-resistant *Staphylococcus aureus* (ORSA) infections with a vancomycin MIC of 1–2 mcg/mL [17-18]. Moreover, an AUC/MIC ≥ 400 has been shown to correlate best with clinical success. As such, recommendations for therapeutic drug monitoring of vancomycin has been published by the American Society of Health-System Pharmacists (ASHP), Infectious Disease Society of America (IDSA), and Society of Infectious Disease Pharmacists (SIDP) [19-22]. However, guidelines specific to the HD patient population are lacking and thus most healthcare facilities and systems have traditionally resorted to adapting the general guidelines to HD patients. Adaptation of these guidelines addresses, in some regard, the renal clearance but does not account for the non-renal clearance of vancomycin. As such, adapted vancomycin dosing protocols usually involve administering a dose after or during the last hour of dialysis. Given the lack of a standardized guideline for this patient population, therapeutic monitoring is of great importance to ensure appropriate doses are administered in order to acquire therapeutic serum concentrations [9]. Thus, while there is a plethora of literature to suggest dosing regimens in adults with normal renal function, there is still a degree of uncertainty as to what the proper dosing regimen would be in this hemodialysis patient population. The goal of this study is to determine if a pre-dialysis vancomycin serum concentration of 15–20 mcg/mL is associated with positive outcomes in patients undergoing intermittent hemodialysis (IHD) with Gram-positive bacteremia.

## Materials and methods

**Figure 1:**
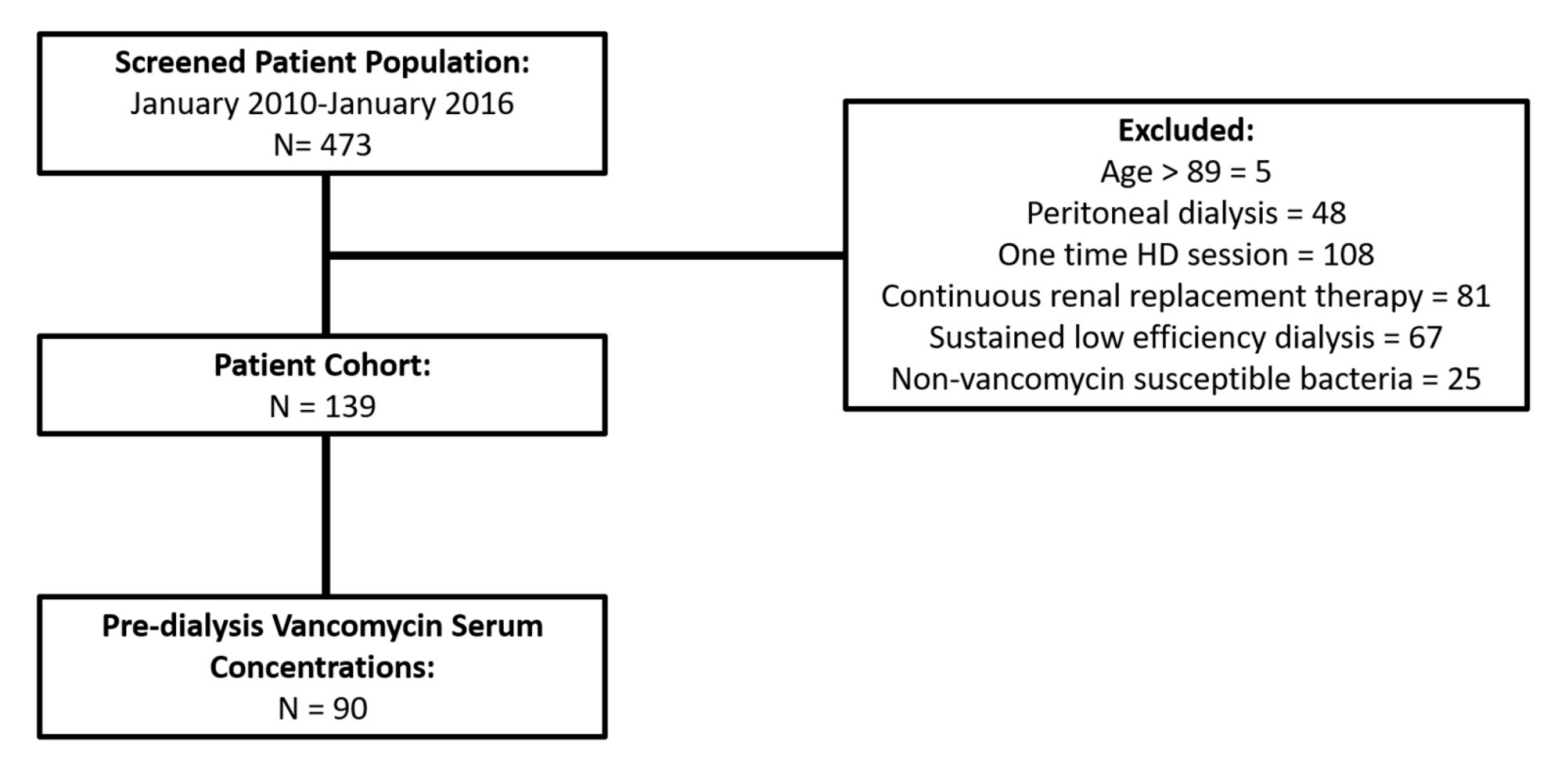
Flowchart of patient selection.

This retrospective cohort study was conducted at a 443-bed tertiary teaching county hospital from January 1, 2010 to January 1, 2016. The year 2010 was selected as the start of the data collection since this was one-year post-implementation of online documentation thus allowing for an adjustment time period. Patients were identified using the International Classification of Diseases, Ninth Revision, Clinical Modification (ICD-9 CM) code for bacteremia (R790.7) and/or the International Classification of Diseases, Tenth Revision, Clinical Modification (ICD-10 CM) code (R78.81). According to the criteria of Tabachnick and Fidell (n ≥ 50 + 8 m, where m is the number of independent variables in the model [m = 2]), the sample size of 139 patients was sufficient to avoid a type II error [23-24]. Inclusion criteria consisted of patients aged 18-89, with chronic renal failure (stage 5 chronic kidney disease) on intermittent hemodialysis who presented with positive blood cultures with Gram-positive bacteria, received vancomycin for at least 24 hours, and were admitted to the intensive care unit (ICU). All patients were treated with high flux polysulfone Optiflux F160NR dialyzers (1.5 m2) (Fresenius Medical Care North America). Exclusion criteria consisted of patients who had an allergic reaction or hypersensitivity to vancomycin, were pregnant, received peritoneal dialysis, had only a one-time hemodialysis session for toxicity, received only one dose of vancomycin, were on continuous renal replacement therapy or sustained low-efficiency dialysis, or had non-vancomycin susceptible bacteria (Figure 1). Age, sex, dry weight, the first pre-dialysis vancomycin serum concentration, organism(s), loading dose, length of dialysis session, mortality, and length of stay were collected. The wet weight (weight upon admission) was utilized for vancomycin dosing. Simplified Acute Physiology Score II (SAPS II) scores were calculated upon admission to the ICU, post the vancomycin loading dose, and utilized as the predictor of mortality. This study was approved by the appropriate institutional review board.

Bacteremia was defined as the occurrence of two consecutive positive blood cultures in the same set drawn on the same day and therapy was adjusted accordingly once susceptibility results were obtained [25]. Clinical cure was defined as a complete resolution of clinical signs and symptoms of infection (fever, leukocytosis, local signs of infection, and negative blood cultures) and mortality was defined as 30-day mortality. Pre-dialysis levels were drawn prior to the IHD session following the loading dose. The vancomycin loading dose was determined by the physician. For levels less than 15 mcg/mL post HD, 500–1000 mg was given.

Statistical analyses were performed using Analyse-it® for Microsoft Excel 3.90.7 (Copyright 1997-2016, Leeds, England). Descriptive statistics were used to compare patient demographics. Nominal data were compared using Pearson’s chi-squared or Fisher’s exact test. Continuous data were analyzed using the Shapiro-Wilk test for normality. In the case of non-parametric data, the median was used for central tendency with interquartile range (IQR) for the dispersion. The Mann-Whitney U test and Spearman’s correlation were used as appropriate. A multivariate analysis was utilized comparing factors related to outcomes including SAPS II score, vancomycin dose, and in-hospital mortality. Factors that had a p-value < 0.02 were kept in the model and the multivariate analysis was repeated. This was executed for each outcome variable. An alpha level of significance was defined a priori as <0.05.

## Results

**Figure 2:**
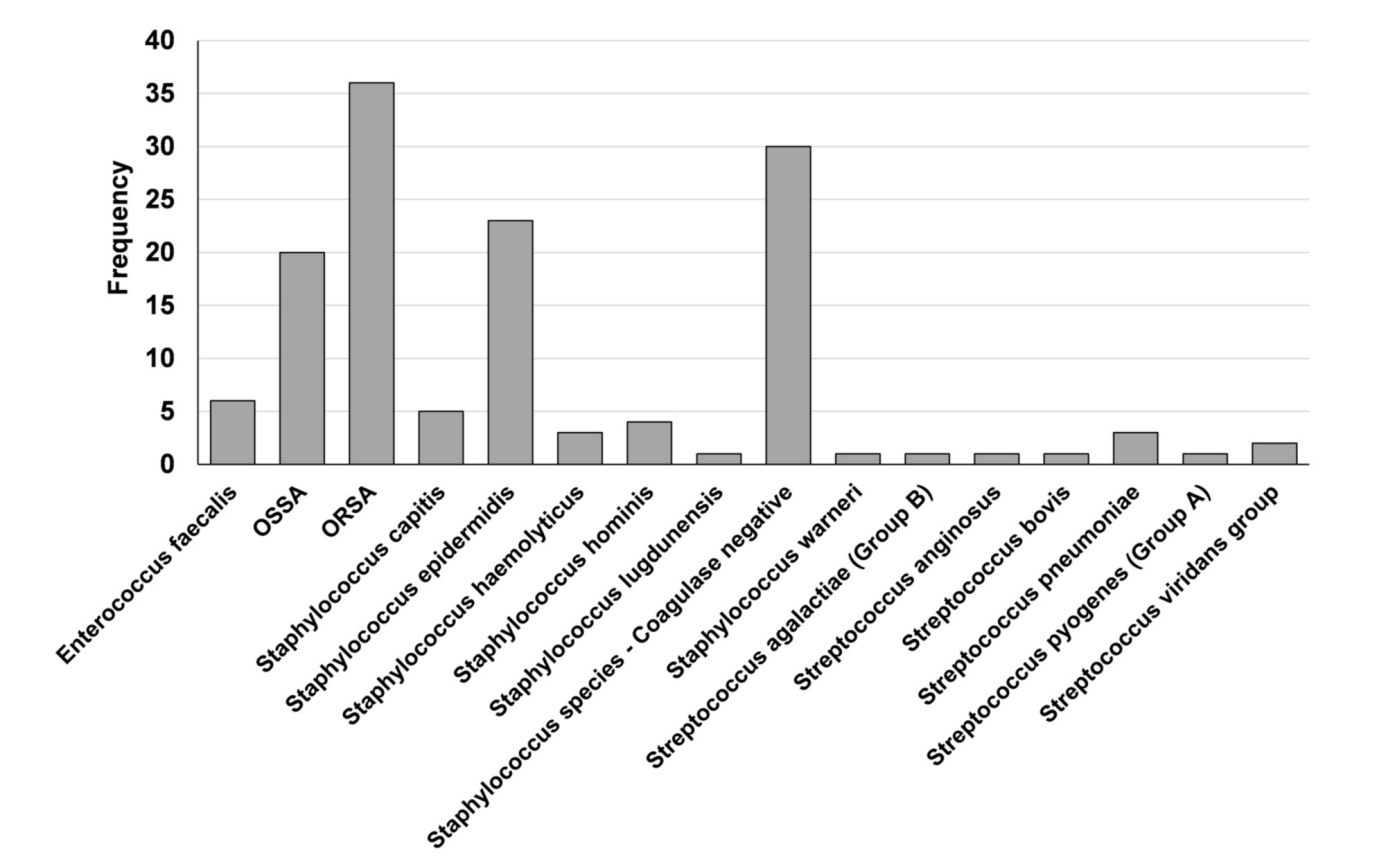
Frequency of blood culture organisms (N = 90). ORSA: Oxacillin-resistant *Staphylococcus aureus*; OSSA: Oxacillin-sensitive *Staphylococcus aureus*.

The studied patient cohort consisted of 139 ESRD patients receiving IHD (76 male, 63 female; median (IQR) age of 58 years (IQR 21.1)). Median weight was 77.1 kg (IQR 30.7 kg). The majority of patients presented with infections of ORSA (MIC < 2 mg/mL), *Staphylococcus epidermidis*, coagulase-negative staphylococci, and/or oxacillin sensitive *Staphylococcus aureus* (OSSA) (Figure 2). The average duration of dialysis was 3.7 h (standard deviation (SD) 0.8 h, range 1.0–6.0 h), average dialysate flow rate was 720 mL/min (SD 31.3 mL/min, range 600–800 mL/min), and the average blood flow rate was 386 mL/min (SD 40.0 mL/min, range 270–450 mL/min). Of 139 patients, 90 had documented pre-dialysis serum vancomycin concentrations. The median (IQR) pre-hemodialysis serum concentration of vancomycin was 16.3 mcg/mL (9.28) (Table 1). Of the 90 patients, 28 had pre-dialysis concentrations less than 15 mcg/mL and 27 had pre-dialysis concentrations more than 20 mcg/mL and 26 patients had a pre-dialysis concentration between 15 and 20 mcg/mL.

**Table 1:** Population baseline characteristics (N = 90). HD: Hemodialysis; IQR: Interquartile range; SAPS-II: Simplified Acute Physiology Score II; SD: Standard deviation.

Characteristics	Patient Sample
Median (IQR) age	58 (21.1)
Sex (M/F)	47/43
Median (IQR) weight in kg	77.1 (30.7)
Median (IQR) SAPS-II score	35 (14.0)
Mean ± SD dialysate flow rate	720 mL/min ± 31.3
Mean ± SD duration of HD	3.7 hr ± 0.8
Mean ± SD blood flow rate	386 mL/min ± 40
Median (IQR) pre-HD vancomycin serum concentration	16.3 mcg/mL (9.28)

For the entire sample size of 139 patients, the median SAPS II score was 35 (IQR 15.0). For the 26 patients achieving the pre-dialysis serum concentration of 15–20 mcg/mL, the median SAPS II score was 34.5 (IQR 12.3) compared to 35.5 (IQR 14.0) in those that did not achieve that serum concentration (p = 0.9). The median (IQR) SAPS II scores were significantly higher in patients who died [47 (8.2)] than those patients who survived [34 (12.2)] (p < 0.0001). Median (IQR) loading doses of vancomycin were lower in patients who died [11.60 mg/kg (3.51)] versus those who survived [13.79 mg/kg (5.51)] (p = 0.09) but did not reach significance on its own. In hospital, mortality was lower in patients who achieved pre-dialysis serum vancomycin concentrations between 15 and 20 mcg/mL (1/26, 3.8%) versus those who did not achieve a pre-dialysis serum concentration between 15 and 20 mcg/mL (11/64, 17.2%) but did not reach significance (p = 0.09) on its own (Tables 2, 3). A multivariate analysis showed that SAPS II score [OR 1.220 (95% CI: 1.086–1.370, p < 0.0001)], loading dose/kg [OR 0.7911 (95% CI: 0.6302–0.9929, p = 0.02)], and pre-dialysis serum vancomycin concentrations between 15 and 20 mcg/mL [0.05437 (95% CI: 0.0033–0.8891, p = 0.01)] were associated with decreased mortality (overall multivariate model, p < 0.0001) (Table 4). Patients who achieved a clinical cure had a significantly higher median (IQR) pre-dialysis vancomycin serum concentration of 18.90 mcg/mL (19.28) than those patients who did not achieve a clinical cure 16.20 mcg/mL (9.57) (p = 0.04).

**Table 2:** Univariate analysis of mortality (N = 90). IQR: Interquartile range; SAPS II: Simplified Acute Physiology Score II.

Parameter	Alive Patients	Deceased Patients	p-Value
Median (IQR) SAPS II	34 (12.2)	47 (8.2)	0.0001
Median (IQR) loading vancomycin dose	13.79 mg/kg (5.51)	11.6 mg/kg (3.51)	0.09

**Table 3:** Univariate analysis of mortality (N = 90).

Parameter	Achieved pre-dialysis vancomycin serum concentration between 15 and 20 mcg/mL		p-Value
Mortality	Yes = 1/26 (3.8%)	No = 11/64 (17.2%)	0.09

**Table 4:** Multivariate analysis of mortality (N = 90); overall model p SAPS II: Simplified Acute Physiology Score II.

Parameter	Odds Ratio	95% CI	p-Value
SAPS II score	1.22	1.08 to 1.37	<0.0001
Loading dose mg/kg	0.79	0.60 to 0.99	0.0239
Pre-dialysis vancomycin level of 15–20 mcg/mL	0.054	0.0033 to 0.89	0.0099

## Discussion

The findings presented here demonstrated a significant association between a pre-dialysis serum concentration of 15–20 mcg/mL and reduced mortality in patients receiving intermittent hemodialysis. The study did not show any statistically significant differences in mortality or relapse/recurrence rates with various post-dialysis vancomycin serum concentrations. Based on the multivariate analysis, both loading dose and pre-dialysis serum vancomycin concentrations had a larger impact on mortality in patients with higher acuity.

Unlike patients with normal renal function, hemodialysis patients have multiple factors that need to be considered, such as dialysis modality and type, timing of vancomycin administration, target serum concentrations, and pharmacokinetic factors. Studies have suggested using loading doses of 15–20 mg/kg followed by maintenance doses of 500–1500 mg [6,26]. In a three-way randomized crossover trial conducted by Mason et al., a loading dose of 15 mg/kg resulted in a pre-hemodialysis vancomycin serum concentration of 23.8 mcg/mL ± 4.8 two days later [27]. Conversely, Barth and DeVincenzo reported the pre-hemodialysis vancomycin serum concentrations two days after a loading dose of 15 mg/kg and 20 mg/kg were 12.6 mcg/mL and 16.3 mcg/mL, respectively [6]. It should be noted that this patient population has multiple factors that can affect the achievement of ‘trough’ levels of 20 mcg/mL. Thus whilst some studies may be able to achieve this level with a loading dose of 12–20 mg/kg, it may not be possible in other studies as the patient specific dynamic affects the results. Hence using a one size fits all dosing regimen may potentiate the risk for underdosing or overdosing these patients. One could argue that higher loading doses can potentiate toxicity; however, nephrotoxicity in this patient population is negligible as approximately 30–50% of vancomycin is removed in one dialysis session [13-16]. Literature has shown there is a rebound in plasma vancomycin concentrations approximately 3–6 hours after high flux dialysis, thus drawing a trough level immediately after dialysis would lead to a gross over-estimation in dialytic clearance [28]. One way to circumvent this overestimation would be to look at pre-HD levels. Several studies have shown dosing based on pre-HD levels achieves therapeutic concentrations, though the endpoint of mortality has not been fully explored [29-30]. Theoretically, if the pre-HD levels are therapeutic, then it could be postulated there may be an improvement in mortality. Additionally, the current literature recommends using weight-based doses (loading and maintenance) in patients receiving vancomycin; however, there is no consensus guideline as to which weight (i.e., wet or dry, actual or ideal) should be used for dosing in hemodialysis patients. Moreover, the protocol at the time of this review placed emphasis on administering a maintenance dose four hours post dialysis. Additionally, whilst the protocol was not specific to pre-dialysis level drawing, the levels obtained were drawn three hours before the next dialysis session. Since there is no consensus guideline on when to draw levels and administer the dose, our protocol does not significantly impact our findings. In our patient population, the wet weight was used for dosing purposes and may be a point of contention. Interestingly, lower pre-dialysis serum concentrations were more prevalent in patients who were ≥61 years. This study was not designed to look at the relationship between dosing, serum concentrations and age; however, this should be explored further in this population.

The results obtained in this study may be generalizable to the hemodialysis population; however, this study has several limitations. There was no specification as to when the loading dose was given before the first dialysis session or at the end of the first dialysis session. Secondly, the patients included were already established on hemodialysis without consideration for any residual renal function. Finally, patients may have received a dose of vancomycin prior to presenting to our institution. As such, these doses were not accounted for, nor were they present in the documentation. Of note, two consecutive positive blood cultures were obtained, which theoretically may be unnecessary for organisms like *Staphylococcus aureus* given the risk involved in this patient population. However, given this patient population is predisposed to infections, treatment was initiated until the second culture result was available.

## Conclusions

When patient acuity and loading dosing are taken into account, pre-dialysis vancomycin serum concentrations between 15 and 20 mcg/mL were associated with a decrease in mortality in Gram-positive bacteremic IHD patients. Further prospective studies are needed to assess whether these targeted serum vancomycin concentrations improve mortality.
